# Prolactin-Releasing Peptide: Physiological and Pharmacological Properties

**DOI:** 10.3390/ijms20215297

**Published:** 2019-10-24

**Authors:** Veronika Pražienková, Andrea Popelová, Jaroslav Kuneš, Lenka Maletínská

**Affiliations:** 1 Biochemistry and Molecular Biology, Institute of Organic Chemistry and Biochemistry of the Czech Academy of Sciences 16610 Prague, Czech Republic; prazienkova@uochb.cas.cz (V.P.); andrea.popelova@uochb.cas.cz (A.P.); kunes@biomed.cas.cz (J.K.); 2 Experimental Hypertension, Institute of Physiology of the Czech Academy of Sciences, 14200 Prague, Czech Republic

**Keywords:** prolactin-releasing peptide, GPR10, RF-amide peptides, food intake regulation, energy expenditure, neuroprotection, signaling

## Abstract

Prolactin-releasing peptide (PrRP) belongs to the large RF-amide neuropeptide family with a conserved Arg-Phe-amide motif at the C-terminus. PrRP plays a main role in the regulation of food intake and energy expenditure. This review focuses not only on the physiological functions of PrRP, but also on its pharmacological properties and the actions of its G-protein coupled receptor, GPR10. Special attention is paid to structure-activity relationship studies on PrRP and its analogs as well as to their effect on different physiological functions, mainly their anorexigenic and neuroprotective features and the regulation of the cardiovascular system, pain, and stress. Additionally, the therapeutic potential of this peptide and its analogs is explored.

## 1. Introduction

There is no doubt that the function of prolactin-releasing peptide (PrRP) in organisms is quite important as its structure is well conserved within different animal species. PrRP is reported to regulate food intake and energy metabolism, but it could have several other specific functions, such as the regulation of cardiac output, stress response, reproduction, the release of endocrine factors, and recently neuroprotective features. The site of the main action of PrRP is the brain, where its release is regulated by a number of stimuli, including those coming from the periphery.

PrRP binds with high affinity to the GPR10 receptor and also has lesser activity towards the neuropeptide FF (NPFF) receptor type 2 (NPFF-R2). In addition, cooperation with other food intake regulating neuropeptides, especially leptin, cholecystokinin (CCK), or neuropeptide Y (NPY), is very important for the effects of PrRP.

In structure-activity relationship (SAR) studies, novel PrRP analogs with attached fatty acids and changes in the amino acid chain were synthetized to overcome the blood-brain barrier and to improve the stability and bioavailability from the periphery, thus representing interesting targets for therapeutic use.

## 2. Discovery and Structure of PrRP

PrRP was first isolated in 1998 by Hinuma and colleagues from an extract of bovine hypothalamus and was described as a ligand for the orphan seven-transmembrane-domain receptor (7TM) GPR10 (also known as hGR3 or rat ortholog UHR-1) using reverse pharmacology ([1,2] and reviewed in [3]). The cloned full-length cDNA of the *PrRP* gene is 435 bp in length and encodes an 87 amino acid long precursor [4]. The *PrRP* rat gene contains three exons and two introns and spans a region of approximately 2.4 kb [5].

The average precursor length is 105 amino acids with two cleavage sites [6]. From the protein precursors, at least two isoforms of different lengths, PrRP20 and PrRP31 (Table 1), are produced. Shorter PrRP20 shares identical C-termini with the longer form of PrRP31. The fish ortholog of PrRP20, C-RFa, was isolated and described by Fujimoto et al. from the brain of *Carassius auratus langsdorfii* in the same year that PrRP was discovered [7]. The cloned cDNA of the *C-RFa* gene is 997 bp in length and encodes a precursor of 108 amino acids [4]. Subsequently, PrRP was identified in amphibians in *Xenopus laevis* in both isoforms [8]. In birds, specifically in *Gallus gallus*, PrRP has a similar sequence as in fishes and amphibians and is also expressed in the brain [9]. Moreover, Wang et al. measured the expression of both PrRP and C-RFa in chickens, as well as in *Xenopus* and zebrafish, suggesting that those peptides are encoded by two separate genes and may play similar yet distinctive roles in nonmammalian vertebrate species [4].

PrRPs in vertebrates share very conserved homology and there is evidence that PrRP evolved from a common ancestry precursor in nonmammalian and mammalian species [10]. The precursor is composed of a hydrophobic N-terminal sequence, paired basic amino acids for the recognition of endopeptidases, and a very conserved C-terminal sequence, where the amino acid glycine is a donor for the amide group. The bovine/human C-terminal octapeptide is Gly-Ile-Arg-Pro-Val-Gly-Arg-Phe-NH_2_; in fish C-RFa, isoleucine and valine are swapped (Table 1) [6].

The name of PrRP was suggested on the basis of its prolactin-releasing activity in a rat pituitary adenoma-derived cell line and in pituitary cells obtained from lactating rats [1]. Additionally, another study reported that the injection of PrRP stimulated plasma prolactin levels in female rats in proestrus, estrus, and metestrus, and increased doses of PrRP were necessary to increase plasma prolactin in male rats [11]. Nevertheless, this prolactin-releasing function was later questioned because it did not have typical features for hypophysiotropic hormones [12,13]. Currently, PrRP is considered likely to be an anorexigenic (i.e., food-intake-lowering) neuropeptide, which mainly plays a role in the regulation of food intake and energy expenditure [12,14,15,16], but also regulates stress [17,18], sleep [19,20], and the cardiovascular system [21,22,23]. In addition, its potential neuroprotective properties have been described [24,25,26].

## 3. GPR10 Discovery and Gene Location

Using polymerase chain reaction (PCR), Marchese et al. discovered genes encoding novel G-protein coupled receptors (GPCRs), including the human gene for *GPR10* [27]. GPR10 shares high amino acid identity with NPY receptor 1 (NPY-1R) and orphan receptor induced by glucocorticoids (GIR) [27]. The overall amino acid identity is 31% and 46% in the transmembrane domains for NPY-1R and 30% and 46% in those for GIR. This GPR10 receptor was later confirmed to be identical to orphan hGR3 reported as a receptor for PrRP by Hinuma et al. [1]. Human GPR10 shares high homology (89%) with rat ortholog UHR-1 [2]. The human 1107 bp long gene for *GPR10* is located on chromosome 10 q25.3–q26.1 and a related sequence on chromosome 13 q14.3–q21.1, encoding a 370 amino acid long protein [27].

In nonmammalian vertebrates, fish and chicken PrRP receptor genes are located on chromosome 17 and chromosome 5, respectively [27,28]. GPR10 is well conserved in mammals with more than 90% identity, however in chickens, it is only 54% identical compared with the mammalian counterpart, probably because of phylogenetic differences. The most conserved sequence is on the C-terminus of the receptor, particularly the last six amino acid peptides that could interact with a ligand [29,30]. Both isoforms PrRP20 and PrRP31 bind with high affinity to the GPR10 receptor and rat UHR-1 [31].

Later, it was discovered that PrRP has an affinity for NPFF-R2 [32]. Different studies confirmed the molecular and functional identity of the HLWAR77 receptor, which is a common target for NPFF and neuropeptide AF (NPAF), with NPFF-R2 [33]. Human NPFF-R2 shares 89% amino acid identity with its rat ortholog, high homology with NPY receptors [34], and 37% homology with the orexin-A receptor [33].

## 4. Distribution of PrRP and its Receptor GPR10

### 4.1. Distribution of PrRP

**Figure 1:**
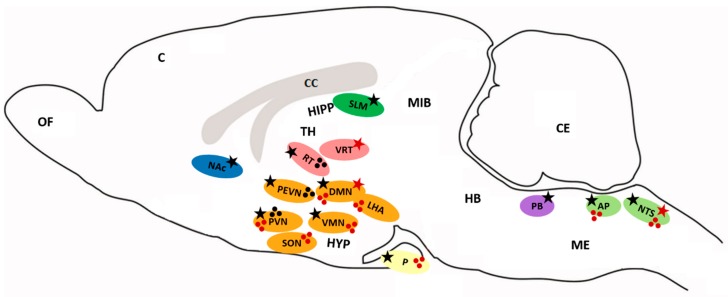
Distribution of PrRP and GPR10. Ellipses represent distinct brain areas (blue—nucleus accumbens, grey—corpus callosum, green—hippocampus, red—thalamus, orange—hypothalamus, yellow—pituitary, violet—parabrachial nucleus, light green—medulla oblongata). Stars mark the expression of mRNA (red star—*PrRP*, black star—*GPR10*). Spots represent the distribution of PrRP (red), GPR10 (black) cell bodies and fibers. AP: area postrema, C: cerebral cortex, CC: corpus callosum, CE: cerebellum, DMN: dorsomedial hypothalamic nucleus, HB: hindbrain, HIPP: hippocampus, HYP: hypothalamus, LHA: lateral hypothalamic area, ME: medulla oblongata, MIB: midbrain, NAc: nucleus accumbens, NTS: nucleus of the solitary tract, OF: olfactory bulb, P: pituitary, PB: parabrachial nucleus, PEVN: periventricular hypothalamic nucleus, PVN: paraventricular hypothalamic nucleus, RT: reticular nucleus of the thalamus, SON: supraoptic nucleus, SLM: stratum lacunosum-moleculare, TH: thalamus, VMN: ventromedial hypothalamic nucleus, VRT: ventrolateral reticular nucleus of the thalamus.

The highest expression of *PrRP* mRNA was measured in the brainstem in the nucleus of the solitary tract (NTS) and a moderate level was detected in the dorsomedial hypothalamic nucleus (DMN), ventrolateral reticular nucleus of the thalamus (VRT) (Figure 1), and in the periphery in the intestine both in rats and humans when analyzed with reverse transcription-PCR [31,35,36]. Immunoreactive cell bodies were found mainly in the DMN, ventromedial hypothalamic nucleus (VMN), NTS, and ventrolateral medulla oblongata (ME), and nerve projections were present in the paraventricular hypothalamic nucleus (PVN), supraoptic nucleus (SON), DMN, lateral hypothalamic area (LHA), thalamic nucleus, amygdala, and area postrema (AP) (Figure 1) [31]. Immunoreactive fibers were also detected in high concentrations in the posterior pituitary [37,38]. Using enzyme immunoassay for PrRP distribution, immunoreactive PrRP was widely present in the hypothalamus, midbrain and posterior pituitary, and ME [37]. In mammals, rats, and humans, peripheral tissue *PrRP* mRNA was found mainly in the adrenal gland, lung, pancreas, liver, kidney, reproductive organs, and gut [35,37,39,40]. Concentration of PrRP in rat plasma was very low (0,13 fmol/mL) [37]. In chicken tissue, *C-RFa* mRNA was detected in the kidney, lung, reproductive organs, heart, intestine, liver, and pituitary [4]. In the amphibious fish, mudskipper, *PrRP* mRNA expression was observed in the brain, liver, gut, and ovary, with lower levels detected in the skin and kidney [41].

### 4.2. Distribution of GPR10

The highest expression of *GPR10* mRNA was detected in several parts of the rat brain, mainly in the reticular nucleus of the thalamus (RT), PVN, periventricular hypothalamic nucleus (PEVN) and DMN, AP, and NTS. A moderate level of expression of the receptor was also detected in the anterior pituitary and VMN (Figure 1) [31,42]. Radiolabeled ^125^I-PrRP31 bound in a specific pattern to the reticular thalamic nucleus and PEVN [31]. GPR10 was also found in the parabrachial nucleus (PB) or nucleus accumbens (NAc), which are areas that are involved in pain processing [31], and in low levels in the hippocampus (stratum lacunosum-moleculare; SLM), which involves areas that are involved in memory [2,26]. In the periphery, *GPR10* mRNA was found in the rat adrenal medulla [35,43,44]. Through the detection of mRNA and in situ hybridization or immunohistochemical studies, PrRP and its receptor were found in discrete areas within the brain and periphery. Indeed, PrRP nerve fibers are in close proximity to areas where GPR10 is present, but PrRP still has to be transported to other sites to be released. This fact may also support the hypothesis that PrRP binding and signaling are not restricted to the GPR10 receptor.

## 5. PrRP Intracellular Signaling Pathways

**Figure 2:**
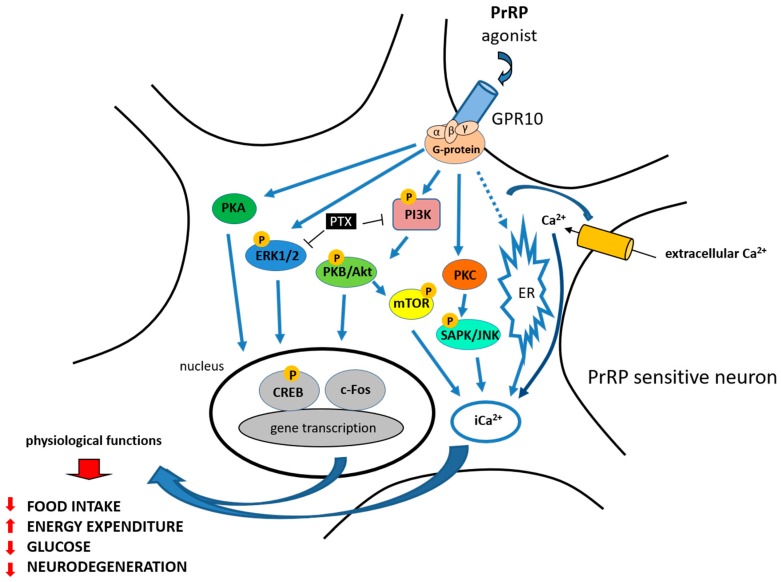
PrRP physiological functions and signaling—summary. PrRP and its agonist exerts its effect through GPR10. Blue arrow represents activation of the signaling pathway, T-bar represents blocking of the signaling pathway. PrRP stimulated calcium release (Ca^2+^) in calcium mobilization assay and rapidly activated extracellular signal-regulated protein kinase (ERK – blue). It also activated c-Jun N-terminal protein kinase (JNK—light blue) and phosphorylated cAMP response element-binding protein (CREB—grey). Pertussis toxin (PTX—black) blocked the ERK and Akt activation induced by PrRP. PrRP activated the PI3K B/Akt-mammalian target of rapamycin (PI3K-Akt-mTOR) pathways in leiomyoma cells (PI3K—pink, PKB/Akt—green,.mTOR—yellow). PrRP significantly stimulated both the PKA (dark green) and PKC (orange) pathways.

To explore signal transduction pathways and the potential agonist or antagonist properties of PrRP action at GPR10, several studies have been published. Hinuma et al. first reported that PrRP promoted arachidonic acid metabolite release in Chinese hamster ovary (CHO) cells expressing GPR10 [1]. PrRP was able to dose-dependently stimulate calcium release in cells that were transfected with GPR10 in a calcium mobilization assay (Figure 2) [31].

PrRP rapidly activated extracellular signal-regulated protein kinase (ERK) from the mitogen-activated protein kinase (MAPK) family in GH3 rat pituitary tumor cells and in primary rat anterior pituitary cultures (Figure 2) [45]. Moreover, pertussis toxin (PTX), which inactivates Gi/Go proteins, completely blocked the ERK activation induced by PrRP, suggesting that at least part of the coupling of GPR10 is through Gi/Go proteins [45]. Kimura et al. also demonstrated that PrRP activated c-Jun N-terminal protein kinase (JNK) in a protein kinase C (PKC)-dependent manner in GH3 rat pituitary tumor cells [45].

PrRP20 was then reported not to alter basal levels of intracellular cyclic AMP in human embryonic kidney HEK293 cells that were transfected with GPR10, suggesting that in this system, GPR10 does not couple through Gs protein, which would activate adenylyl cyclase to increase the cyclic AMP concentration [46]. In addition, PrRP20 did not decrease forskolin-stimulated cyclic AMP levels, indicating that GPR10 does not couple via Gi, which would inhibit adenylyl cyclase and decrease cyclic AMP levels [46]. Therefore, the possible involvement of GPR10 signaling through the Gq pathway was proposed.

Engstrom et al. tested the ability of PrRP20 or PrRP31 to stimulate [^35^S]GTPγS binding to membranes of CHO cells expressing GPR10; more than 80% of the binding of PrRP was prevented by PTX [32]. Taken together, these data suggest that a large part of the GPR10 coupling occurs via Gi/Go proteins, however this depends on the cellular system in which the receptor is expressed [32,45,46]. In the study from Engstrom et al., intracellular calcium assays also confirmed the full agonist properties of both PrRP20 and PrRP31 at GPR10 [46].

PrRP rapidly and transiently stimulated the activation of protein kinase B (Akt) in GH3 cells, and a phosphoinositide 3-kinase-protein kinase (PI3K) inhibitor blocked the PrRP-induced activation of Akt (Figure 2) (reviewed in [47]). Additionally, PTX completely blocked the Akt activation induced by PrRP, suggesting the involvement of Gi/Go proteins [48]. PrRP31 significantly induced an increase in the activity of ERKs and JNK, but not p38 MAPK in the rat PC12 pheochromocytoma cell line [49]. Moreover, PrRP stimulated dopamine release and catecholamine secretion and increased tyrosine hydroxylase levels via the protein kinase A (PKA) and PKC pathway in PC12 cells [49,50]. PrRP has also been shown to stimulate adenylyl cyclase in the PC12 cell line and promote the proliferation of cultured cells [51]. The stimulation of the chicken PrRP receptor expressed in CHO cells by PrRP also leads to the activation of the intracellular PKA signaling pathway [4,52].

PrRP activated the PI3K B/Akt-mammalian target of rapamycin (PI3K-Akt-mTOR) pathways and cell proliferation in primary leiomyoma cells, where GPR10 is aberrantly expressed [53]. Maixnerova et al. showed that both PrRP20 and PrRP31 activated ERK and cAMP response element-binding protein (CREB) signaling and induced prolactin release in the rat pituitary cell line RC-4B/C with equal potency (Figure 2) [54]. Additionally, modified analogs of PrRP20 and PrRP31, either with changes in the amino acids at the C-terminus or with lipidization, strongly induced the phosphorylation of the ERK pathway in CHO cells expressing GPR10 [55].

## 6. Structure-Activity Relationship Studies

Two isoforms of PrRP with either 20 or 31 amino acids sharing identical C-termini showed comparable in vitro and in vivo activity [1]. Several SAR studies with PrRP analogs were performed [31,56,57,58]. No study about selective antagonists of PrRP has been published yet, but in 2010, Otsuka Pharmaceuticals patented nonpeptide heterocyclic antagonists derived from tetrahydropyridol [4,3-d]pyrimidinone developed for stress-related diseases (reviewed in [59]).

First, Roland et al. demonstrated that N-terminal deletions from PrRP20 slightly decreased the affinity of the PrRP analogs for GPR10 [31]. The shortest analog that was still able to bind to GPR10 was C-terminal heptapeptide PrRP(25–31). However, this fragment displayed a two order of magnitude decrease in binding affinity compared to that of PrRP20 and PrRP31, which exhibited affinity in the nanomolar range. The replacement of the C-terminal amide group with an acid resulted in a complete loss of binding affinity [1,31]. Moreover, an alanine scan through PrRP(25–31) showed that the arginine at positions 26 and 30 is crucial for binding to the receptor, and their change results in a loss of affinity [31]. D’Ursi et al. described a conformational analysis of PrRP20 using circular dichroism (CD) and nuclear magnetic resonance (NMR) spectroscopies and molecular modeling calculations. The C-terminal region consisted of amphipathic helices with hydrophobic nonpolar side chains of Ala^21^, Ile^25^, Val^28^, and Phe^31^ and hydrophilic side chains of Arg^23^, Arg^26^, and Arg^30^ [60].

PrRP could be shortened without a loss of in vitro activity to the tridecapeptide PrRP(19–31), H-Trp^19^-Tyr^20^-Ala^21^-Ser^22^-Arg^23^-Gly^24^-Ile^25^-Arg^26^-Pro^27^-Val^28^-Gly^29^-Arg^30^-Phe^31^-NH_2_, which has the minimal length for retaining binding affinity and agonist properties [56]. The binding affinity was significantly decreased by further truncation of the peptide; therefore, the active site is located within the C-terminal region. This large SAR study focused on the replacement of amino acids at positions 21 to 31, with a main focus on the phenylalanine at position 31. Nineteen different amino acids were used, but only a bulky side chain His(Bzl), Trp, Cys(Bzl), Glu(Obzl), norleucine (Nle) or a halogenated aromatic ring (Phe(4-Cl)) led to similar or improved binding affinity and good agonist activity [56]. Replacement of Arg^23^ by Pro significantly decreased the affinity. The results confirmed that the functionally important residues are located within the C-terminal segment with the essential and irretrievable arginine 30 and the high importance of phenylalanine 31.

Based on a previous study by Boyle et al., Maletínská et al. [58] designed PrRP20 analogs with modifications of Phe^31^ by amino acids with different aromatic rings. Phe^31^ was replaced by (3,4-dichlor)phenylalanine (PheCl_2_^31^), (4-nitro)phenylalanine (PheNO_2_^31^), pentafluoro-phenylalanine (PheF_5_^31^), napthylalanine (1-Nal^31^, 2-Nal^31^), or Tyr^31^. In addition, the amino acids cyclohexylalanine (Cha^31^) and phenylglycine (Phg^31^) were included [58]. This study showed that all analogs except [Cha^31^]PrRP20 and [Phg^31^]PrRP20 preserved high binding affinity to rat RC-4B/C pituitary cells and increased the phosphorylation of ERK and CREB in this cell line.

DeLuca et al. performed a structural study based on NMR and CD spectroscopy, where they determined the α-helical conformation in trifluoroethanol of the C-terminal sequence of PrRP20 [57]. Shorter PrRP20 analogs, PrRP(4–20), PrRP13 (PrRP(8–20)), and heptapeptide PrRP(14–20), decreased the stability of the helical segment and their biological activity was reduced. Therefore, this stable C-terminal α-helical structure facilitates ligand recognition by the receptor and enables its activation [57].

The lipidization of peptides (i.e., the attachment of fatty acids to peptides through an ester or amide bond) is a useful strategy for designing new peptide drugs. This modification may enhance potency, selectivity, and therapeutic efficacy because it can increase stability and prolong the half-life in an organism. Moreover, it could enable delivery across the blood-brain barrier (reviewed in [61]). This lipidized peptide is liraglutide, an analog of glucagon-like peptide 1 (GLP-1) that is palmitoylated at position 26 via a γ-glutamyl linker [62], with a strongly prolonged half-life [63]. Therefore, the lipidization of neuropeptides that is involved in food intake regulation might be a new way for the development of drugs for the treatment of obesity (reviewed in [64]).

Maletínská et al. designed novel lipidized PrRP analogs with fatty acids of different lengths attached to the N-terminus [55]. All PrRP20 and PrRP31 analogs lipidized with octanoic, decanoic, dodecanoic, myristic, palmitic, and stearic acid had agonist characteristics and preserved high binding affinity to GPR10 compared to native PrRP20 or PrRP31 [55].

Lipidized PrRP31 analogs with noncoded amino acids 1-Nal, PheCl_2_, PheNO_2_, PheF_5_, or Tyr at position 31 and myristoylated or palmitoylated on the N-terminus revealed high binding potency to GPR10. The original methionine at position 8 was replaced by the more stable Nle to avoid oxidation of Met without any loss of binding and signaling activity [65].

Analogs of PrRP31 where palmitic acid was attached through the γ-glutamyl linker or the short chain of polyethylene glycol at Lys^11^ or analog with two palmitic acids at Lys^11^ and at the N-terminus were tested both in in vitro and in vivo studies. Binding and signaling experiments showed preserved binding affinity to GPR10, although the analog with two palmitic acids was less potent. The attachment of the single palmitic acid could be performed on different positions of the chain without the loss of binding affinity [66].

**Figure 3:**
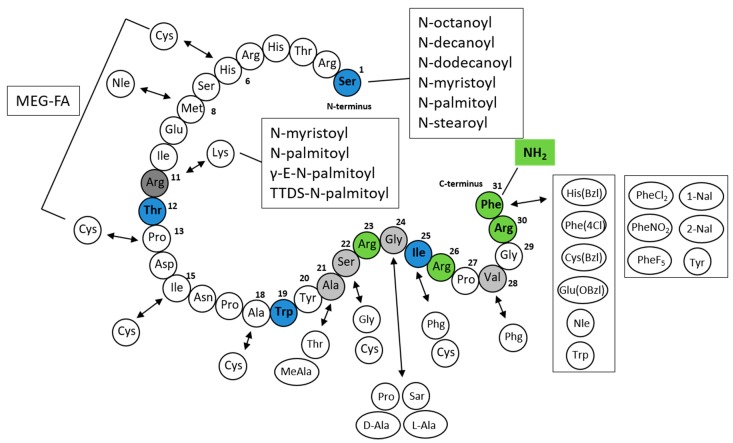
Structure of PrRP20 and PrRP31 and its analogs [31,55,56,58,65,67]. Blue amino acid Ser^1^ marks the beginning of PrRP31 from the N-terminus. Blue amino acid Thr^12^ marks the beginning of shorter isoform PrRP20 (also known as PrRP(12–31)). Blue amino acid Trp^19^ marks tridecapeptide PrRP(19–31) and blue Ile^25^ marks the shortest fragment heptapeptide PrRP(25–31). Green amide group NH_2_ and green Arg^23^, Arg^26^, Arg^30^, and Phe^31^ amino acids are essential for the functionality of the peptide. Light grey amino acids mark changes of amino acids that preserved good functional activity. Dark grey Arg^11^ could be substituted by Lys^11^ and its secondary amino group, fatty acids, were attached through different linkers. γ-E: γ-glutamic acid, MEG-FA: multiple ethylene glycol-fatty acid (four ethylene glycol units attached to octadecanedioic acid via lysine linker incorporating carboxylated moiety), PheCl_2_: (3,4-dichlor)phenylalanine, PheNO_2_: (4-nitro)phenylalanine, PheF_5_: pentafluoro-phenylalanine, 1-Nal, 2-Nal: napthylalanine, Phg: phenylglycine, TTDS: short chain of polyethylene glycol (1,13-diamino-4,7,10-trioxadecan-succinamic acid).

Recently, a new study by Pflimlin et al. was published in which novel long-lasting PrRP analogs with staples incorporating multiple ethylene glycol-fatty acids (MEG-FAs) were synthetized [67]. Crucial arginines at positions 23 and 30 were replaced with homoarginine (hArg), beta-homoarginine (β-hArg), and N-methylarginine (Nme-Arg). All modifications at Arg^30^ significantly affected the potency. In Arg^23^, only substitution by Nme-Arg, but not by hArg or β-hArg, decreased the affinity. All synthetized analogs contained dicysteine mutations, the best tolerated of which occurred at positions 6–13, 15–22, and 18–25. As lead compounds, they chose the PrRP analog 18-S4, an analog with Cys^6^, Cys^13^, Nle^11^, and hArg^23^ and stapled at cysteines by staple featuring four ethylene glycol units attached to octadecanedioic acid via a lysine linker incorporating a carboxylated moiety. They generated analogs with in vitro selective agonist activity towards GPR10 [67]. The structure of all of the mentioned PrRP analogs is described in Figure 3.

## 7. PrRP in the Regulation of Food Intake and Energy Expenditure

### 7.1. PrRP Decreases Food Intake and Regulates Energy Homeostasis

First, it was shown that PrRP caused the release of prolactin from cultured pituitary cells [1], but later, other studies showed the main role of PrRP to be in food intake regulation ([12,14,68] and this is reviewed in [64]).

Lawrence et al. suggested an alternative role for PrRP as a regulator of energy homeostasis and food intake [14]. Intracerebroventricular (ICV) injection of PrRP caused a reduction in food intake in fasted and free-fed rats [14]. Moreover, the subsequent decrease in body weight was not only due to the reduction in food intake, which implies an effect on energy expenditure. They supported the findings by measuring *PrRP* mRNA, which was highly expressed in the hypothalamus, NTS, and ventrolateral ME, and *GPR10* mRNA in the RT, PEVN and DMN, and NTS, all areas of which are implicated in the regulation of food intake (reviewed in [69,70,71]).

PrRP also mediated some of the central satiating actions of the gut peptide hormone CCK [12]. The measurement of the induction of c-Fos protein showed that PrRP neurons were strongly activated by the intraperitoneal injection of CCK, and central PrRP administration activated areas of the brain that are common for both PrRP and CCK [12]. Ellacott et al. suggested that the anorexigenic action of PrRP is regulated by the adiposity signal leptin [72]. ICV administration of PrRP and leptin resulted in reduced food intake in rats and an increase in body temperature compared with each peptide alone. Additionally, using in situ hybridization, *PrRP* mRNA levels were reduced in fasting and obese Zucker rats, indicating that *PrRP* expression is regulated by leptin [72].

Repeated ICV injection of PrRP strongly reduced food intake and body weight in rats without causing any adverse behavior on locomotor or sensor motor activity [73]. PrRP exerted an effect on energy homeostasis in the short to medium term and increased energy expenditure [74].

Through the generation of *GPR10* knockout (KO) mice with targeted deletion of the *GPR10* gene, GPR10 was confirmed to be a major receptor for PrRP in the hypothalamus because this deletion completely prevented PrRP binding to hypothalamic cell membranes [75]. *GPR10* KO mice become hyperphagic and mildly obese at older ages and develop decreased glucose tolerance with elevated levels of insulin and leptin [75]. Male and female *GPR10* KO mice had increased body weight as a consequence of increased fat mass compared to their wild-type (WT) controls [76]. The total levels of plasma leptin and cholesterol were increased, and a decrease in energy expenditure was observed in *GPR10* KO mice [76]. In fasted or satiated *GPR10* KO mice, ICV administration of PrRP did not reduce food intake in contrast to their WT controls. The administration of CCK did not result in the inhibition of food intake in *GPR10* KO mice, suggesting that PrRP is involved in central satiating actions of CCK [77]. *PrRP* KO mice had higher blood glucose levels and corticosterone levels and became obese with higher amounts of adipose or liver tissue than control WT animals [78]. Under stress conditions, *PrRP* KO mice showed increased levels of plasma corticosterone compared to WT mice, which might indicate that PrRP regulates glucose metabolism through corticosterone secretion and⁄or catecholamine synthesis [78].

PrRP was also shown to mediate its anorexigenic effect through corticotropin-releasing hormone (CRH) receptors, but not through melanocortin receptors [68]. ICV administration of PrRP elevated adrenocorticotropin (ACTH) levels in plasma, and c-Fos protein was increased in the nuclei of CRH-positive cells in the PVN [79,80]. PrRP-positive neurons have synapse-like contact with CRH cell bodies in the PVN [79]. Furthermore, the injection of PrRP directly into the PVN caused an increase in plasma ACTH [81]. Using hypothalamic explant incubations, researchers showed that PrRP increased hypothalamic CRH release, which is one of the principal ACTH secretagogues, and the subsequent secretion of ACTH. Therefore, an additional potential role of PrRP in the function of the hypothalamic-pituitary-adrenal (HPA) axis and in the cardiovascular system was suggested [23,81].

### 7.2. Ortholog C-RFa in Food Intake Regulation

Similar to the anorexigenic action of PrRP in mammals, ICV injection of ortholog C-RFa also inhibited food intake in goldfish [82]. However, a completely opposite result was observed in chicks, where ICV injection of rat PrRP31 significantly increased food intake, and the orexigenic effect of NPY was enhanced with the coadministration of PrRP [83]. ICV injection of ortholog C-RFa did not affect food intake in chickens [84].

### 7.3. PrRP Analogs in the Regulation of Food Intake and Energy Expenditure

The C-terminal 20 amino acids of PrRP (PrRP20) are crucial for preserving the full food-intake-lowering effect. ICV administration of PrRP20 analogs with PheCl_2_^31^, PheNO_2_^31^, PheF_5_^31^, 1-Nal^31^, 2-Nal^31^, or Tyr^31^ resulted in decreased food intake in fasted mice [58]. In particular, [PheNO_2_^31^]PrRP20, [1-Nal^31^]PrRP20, [2-Nal^31^]PrRP20, and [Tyr^31^]PrRP20 showed the most significant and long-lasting anorexigenic effect after ICV administration in fasted lean mice. This study showed that a bulky aromatic ring, not necessarily phenylalanine at the C-terminus, enabled full anorexigenic activity [58].

PrRP acts centrally; therefore, the potential of PrRP to decrease food intake after peripheral administration depends on reaching the receptors in the brain and enabling the central effect. Of the analogs with different length fatty acids attached at the N-terminus of PrRP, only myristoylated PrRP20 (myr-PrRP20), palmitoylated (palm-PrRP31), and stearoylated PrRP31 significantly lowered food intake in fasted or freely fed lean mice after subcutaneous (SC) administration [55]. Therefore, those analogs were suggested to probably cross the blood-brain barrier because they caused the central effect after peripheral administration. Analogs containing shorter fatty acids had no effect on food intake. Moreover, analogs palm-PrRP31 and myr-PrRP20, but not natural PrRP20 and PrRP31 or octanoylated PrRP31, showed longer stability in rat plasma and significantly increased c-Fos immunoreactivity in hypothalamic and brainstem nuclei that are involved in food intake regulation, such as PVN, ARC, and NTS.

A significant increase in c-Fos was observed in the PVN, ARC, NTS, and DMN after SC administration of palm-PrRP31. Moreover, palm-PrRP31 administration significantly increased c-Fos in the LHA hypocretin neurons and PVN oxytocin neurons [85].

Palmitoylated or myristoylated PrRP31 analogs with C-terminal changes reduced acute food intake after SC administration in fasted lean mice [65] (reviewed in [64]). Of all the lipidized PrRP analogs, [PheCl_2_^31^]PrRP31 palmitoylated or myristoylated at the N-terminus showed the strongest and long-lasting anorexigenic effect in fasted mice [65]. In free-fed Wistar rats, palm-PrRP31 strongly reduced food intake when injected peripherally. Peripheral injection of palm-PrRP31 induced the increase of c-Fos protein in the PVN, NTS, and ARC, which are specific brain regions that are involved in food intake regulation [86].

In diet-induced obese (DIO) mice, a 2-week-long SC administration of palm-PrRP31 and myr-PrRP20 significantly lowered food intake, decreased body weight, improved metabolic parameters such as plasma insulin and leptin, and attenuated lipogenesis compared to lean controls [55].

Repeated administration of PrRP analogs palmitoylated through different linkers to Lys^11^ but not analog with two palmitic acids reduced body and liver weights and the levels of plasma insulin, leptin, triglycerides, cholesterol, and free fatty acid in DIO mice. Moreover, the expression of *uncoupling protein 1* (*UCP-1*) was increased in brown adipose tissue (BAT), suggesting an increase in energy expenditure [66]. A single dose of PrRP31 palmitoylated at Lys^11^ through a γ-glutamyl linker (palm^11^-PrRP31) again caused neuronal activation and decreased food intake, suggesting its central effect after peripheral administration [66]. This lipidized analog palm^11^-PrRP31 increased the neural activity, represented by increased FosB immunostaining, only in the DMN and in VMN among the analyzed brain nuclei involved in food intake regulation [87].

The chronic effect of palm-PrRP31 was studied in DIO Sprague-Dawley rats and leptin receptor-deficient Zucker diabetic fatty (ZDF) rats, where palm-PrRP31 was intraperitoneally administered for two weeks. Palm-PrRP31 lowered food intake and body weight, improved glucose tolerance, and tended to decrease leptin levels and adipose tissue in DIO rats [88]. In contrast, the administration of palm-PrRP31 lowered food intake, but it did not significantly affect body weight or glucose tolerance in ZDF rats.

Repeated administration of the lipidized PrRP analog palm^11^-PrRP31 improved glucose tolerance in Koletsky-spontaneously hypertensive obese (SHROB) rats, which have mutations in their leptin receptor and, therefore, impaired leptin signaling [89]. These findings suggest that the effect of palm^11^-PrRP31 on glucose metabolism is independent of leptin signaling and body weight lowering. Treatment with palm^11^-PrRP31 also decreased body weight in control spontaneously hypertensive rats (SHRs), but not in SHROB rats. It seems that the palm^11^-PrRP anorexigenic effect depends on the proper leptin signaling. Moreover, in SHROB rats, palm^11^-PrRP31 ameliorated the insulin/glucagon ratio and increased *insulin receptor substrate 1* and *2* expression in fat and insulin signaling in the hypothalamus, while it had no effect on blood pressure [89]. An increase in all parameters mentioned pointed to a beneficial effect of palm^11^-PrRP on the diabetic state. Additionally, in SHRs and normotensive Wistar Kyoto (WKY) rats on a high-fat diet, treatment with palm^11^-PrRP31 lowered body weight and improved biochemical and biometric parameters. Palm^11^-PrRP31 also improved glucose tolerance in WKY rats [90].

Novel long-lasting PrRP analogs with cysteine mutations and staples with attached octadecanedioic acid enhanced plasma stability and half-life in mice. In a 12-day SC administration, the 18-S4 analog significantly reduced body weight in DIO mice [67].

Taken together, PrRP and palmitoylated PrRP analogs are anorexigenic peptides that strongly reduce food intake by reducing appetite and impact energy expenditure under the control of leptin. Proper leptin signaling is necessary for the anorexigenic effect of PrRP and its analogs. Palmitoylated PrRP analogs activate c-Fos in specific neuron populations that are connected to the regulation of food intake. Moreover, lipidization prolonged the half-life of PrRP analogs and enabled central action, leading to a strong food-intake-lowering effect after peripheral administration in mice and rats [55,64,66].

## 8. Neuroprotective Properties of PrRP

Obesity and type 2 diabetes mellitus were recently identified as risk factors for the development of neurological disorders, such as Alzheimer’s disease (AD). Thus, anorexigenic and/or antidiabetic substances began to be examined as compounds with potential neuroprotective properties. This potential is supported by the finding that receptors of anorexigenic peptides, such as GPR10 or the GLP-1 receptor, are expressed in the hippocampus, which is the first brain region affected during AD.

Extracellular senile plaques formed by aggregated β-amyloid protein (Aβ) and intracellular neurofibrillary tangles formed by hyperphosphorylated tau protein are two hallmarks of AD [91,92]. However, other pathological features are observed in AD patients, such as decreased synaptic plasticity and neurogenesis or increased neuroinflammation [93].

The neuroprotective properties of the lipidized PrRP analogs palm-PrRP31 and palm^11^-PrRP31 were examined in vitro as well as in vivo in several rodent models of neurodegeneration. The results were reviewed in depth by Maletínská et al. [94].

The effect of human PrRP31 and its lipidized analog palm^11^-PrRP31 on tau hyperphosphorylation was examined in vitro using a model of hypothermia in the neuroblastoma cell line SH-SY5Y and on rat primary cortical neurons. Hypothermic conditions resulted in increased tau hyperphosphorylation at several epitopes, including pThr212 and pSer396/pSer404, in both cellular models. In SH-SY5Y, incubation with palm^11^-PrRP31, as well as with PrRP31, attenuated tau hyperphosphorylation at pThr212. In primary cortical neurons, palm^11^-PrRP31 decreased tau hyperphosphorylation at both pThr212 and pSer396/pSer404. On the other hand, human PrRP did not affect phosphorylation at pThr212 or at pSer396/Ser404 in primary cortical neurons [95].

The effect of PrRP on tau hyperphosphorylation was extensively studied in vivo using different mouse models. Mice with obesity induced by monosodium glutamate (MSG mice) [96,97] develop increased tau hyperphosphorylation due to central insulin resistance manifested by decreased activation of the insulin signaling cascade. Palm-PrRP31 ameliorated the activation of the insulin signaling cascade and subsequently decreased tau phosphorylation at several epitopes, such as pThr231 and pSer396 [26]. A similar effect on tau hyperphosphorylation was observed in the THY-Tau22 mouse model, where the intervention with palm^11^-PrRP31 also improved short-term spatial memory in the Y-maze test and increased synaptic plasticity compared to the vehicle-treated group [25]. The modulation of synaptic transduction was also examined in a study by Lin et al. [30], where they showed that GPR10 modulates the scaffolding and trafficking of the glutamate-gated cation channel α-amino-3-hydroxy-5-methylisoxazole-4-propionic acid receptor to the postsynaptic membrane, which is necessary to mediate fast excitatory transmission in the brain.

APP/PS1 mice, which are double transgenic mice expressing mutated amyloid precursor protein (APP) (Swedish mutation, K595N/M596L) and mutated presenilin (PS1) (deltaE9 PS1) exon deletion, are one of the most frequently used models to study Aβ pathology [98]. Treatment with the lipidized analog palm^11^-PrRP31 decreased the amount of senile Aβ plaques in APP/PS1 mice. Moreover, palm^11^-PrRP31 lowered the markers of neuroinflammation that are colocalized with Aβ plaques—ionized calcium-binding adapter molecule 1 (Iba1), which is a marker of activated microglial cells, and glial fibrillary acidic protein (GFAP), which is a marker of reactive astrocytes. Potential neuroprotective properties are further manifested by increased levels of doublecortin, a marker of neurogenesis, in hippocampi [24].

In conclusion, palmitoylated analogs of PrRP31 seem to be potential tools to treat neurological disorders. However, the mechanism of action remains unclear and must be further studied.

## 9. Other Physiological Functions of PrRP

*PrRP* and *GPR10* are expressed in many brain regions that control different physiological functions. It seems that PrRP plays an important role in the stress response (reviewed in [99]). PrRP-producing neurons in the ME were activated in response to some stressful stimuli, such as foot shock stress [100]. Moreover, *PrRP* KO mice were found to react differently to restraint stress than their WT littermates; *PrRP* KO mice have increased blood glucose and corticosterone levels [78]. This study was supported by the finding that neurons producing noradrenaline, which is known as a stress mediator in the CNS, are colocalized with PrRP neurons in the NTS and ventral and lateral reticular nuclei in the ME, and coadministration of PrRP and noradrenaline synergistically increased the release of pituitary ACTH [18]. In NTS, PrRP immunopositive neurons are located in close proximity to GLP-1 immunopositive neurons and signaling, though GLP-1R modulates the activity of PrRP neurons [101]. Both neuronal populations are activated after exposure to stressors and seem to contribute to the central control of stress. The PrRP neural populations from ME were projected to the PVN in the hypothalamus, where CRH and oxytocin, both of which are modulators of the stress response, are produced [79]. Consistent with this, ICV administration of PrRP increased the level of corticosterone and oxytocin in the blood. In addition, the administration of PrRP antibodies abolishes stress-induced activation of PVN and attenuated oxytocin release to the blood [102]. The coadministration of PrRP and astressin, a CRH receptor antagonist, blocked ACTH release; thus, the CRH receptor is important for PrRP action [68]. The physiological role of PrRP is well reviewed by Lin [29], Dodd et al. [3], and Quillet et al. [103].

The effect of PrRP on CRH release could be responsible for the increased heart rate and blood pressure that was observed after central PrRP administration [23]; thus, PrRP could be involved in the regulation of the cardiovascular system (reviewed by [22]). It seems that the effect of PrRP on the increase in blood pressure is not mediated by GPR10 since PrRP was able to increase blood pressure in Otsuka Long-Evans Tokushima Fatty (OLETF) rats that have mutated *GPR10* [104].

A high density of GPR10-producing neurons is observed in the PB, which is responsible for the regulation of nociception. These neurons also produced enkephalins, which are pain suppressors that bind to opioid receptors, which suggests the control of enkephalin production by PrRP [105]. The role of PrRP in nociception is supported by the finding that *GPR10* KO mice have a higher nociceptive threshold and increased stress-induced analgesia. Thus, PrRP could act as a potential antagonist of the opioid system [106].

It was also demonstrated that PrRP may affect the function of chromaffin cells because PrRP and its receptor are highly expressed in the adrenal medulla [39,44]. Moreover, PrRP-immunopositive cells were found in the rat adrenal gland [107]. On the basis of these results, it was suggested that PrRP may play an important role in modulating catecholamine secretion [49].

Due to its distribution, PrRP could also be involved in sexual and reproductive function or in sleep and the control of circadian rhythms (in the ME) [19,20]. PrRP is expressed in brain areas that are implicated in reproduction (in the DMN, ME) and also in periphery in rat testis and epididymis [39,59]. Feng et al. suggested that PrRP could be involved in the regulation of the female rat estrous cycle [108]. Brain *PrRP* mRNA level was higher in the proestrus and estrus in female rats. Moreover, they found colocalization of GPR10 immunoreactive neurons and gonadotropin-releasing hormone in the hypothalamic medial preoptic area. The study by Maruyama et al. showed that ICV administration of PrRP increases plasma oxytocin in rats and they suggested the role of PrRP as a neuromodulator of oxytocin neurons in the brain [109]. There is also some evidence that PrRP is involved in lactation and that PrRP levels are regulated by hormonal changes [100].

## 10. Conclusions

PrRP, with its conservative RF-amide sequence on the C-terminus, is a potent anorexigenic neuropeptide, decreasing food intake and enhancing energy metabolism. Moreover, it regulates other physiological functions, such as the cardiovascular system, stress, and reproduction, and has neuroprotective properties. These functions are mainly mediated through the receptor GPR10.

The use of specific model systems, particularly *PrRP/GPR10* KO animals, can contribute to an understanding of the molecular mechanisms of PrRP action, thereby contributing to a faster use of PrRP analogs for potential therapy. From our several recent studies, it is clear that lipidized PrRP analogs could have therapeutic potential. Further progress in the development of selective PrRP analogs may contribute to their use not only in the treatment of obesity, but also in the treatment of other metabolic or neurodegenerative diseases.

## Figures and Tables

**Table 1:** Sequences of prolactin-releasing peptide (PrRP): PrRP20 and PrRP31 in different animal species [1,4,7].

Peptide	Species	Sequence																																						
**PrRP20**	carp																		S	P	E	I	D	P	F	W	Y	V	G	R	G	V	R	P	I	G	R	F	-	NH_2_
	chicken																		S	P	E	I	D	P	F	W	Y	V	G	R	G	V	R	P	I	G	R	F	-	NH_2_
	rat																		T	P	D	I	N	P	A	W	Y	T	G	R	G	I	R	P	V	G	R	F	-	NH_2_
	bovine																		T	P	D	I	N	P	A	W	Y	A	G	R	G	I	R	P	V	G	R	F	-	NH_2_
	human																		T	P	D	I	N	P	A	W	Y	A	S	R	G	I	R	P	V	G	R	F	-	NH_2_
**PrRP31**	carp	G	T	T	V	E	H	D	L	H	I	V	H	N	V	D	N	R	S	P	E	I	D	P	F	W	Y	V	G	R	G	V	R	P	I	G	R	F	-	NH_2_
	chicken							S	R	P	F	K	H	Q	I	D	N	R	S	P	E	I	D	P	F	W	Y	V	G	R	G	V	R	P	I	G	R	F	-	NH_2_
	rat							S	R	A	H	Q	H	S	M	E	T	R	T	P	D	I	N	P	A	W	Y	T	G	R	G	I	R	P	V	G	R	F	-	NH_2_
	bovine							S	R	A	H	R	H	S	M	E	I	R	T	P	D	I	N	P	A	W	Y	A	G	R	G	I	R	P	V	G	R	F	-	NH_2_
	human							S	R	T	H	R	H	S	M	E	I	R	T	P	D	I	N	P	A	W	Y	A	S	R	G	I	R	P	V	G	R	F	-	NH_2_

Grey color marks same amino acids.

## Abbreviations

Aβ	β-amyloid protein
ACTH	Adrenocorticotropin
AD	Alzheimer’s disease
Akt	Protein kinase B
AP	Area postrema
APP	Amyloid precursor protein
BAT	Brown adipose tissue
β-hArg	beta-homoarginine
C	Cerebral cortex
CC	Corpus callosum
CE	Cerebellum
CCK	Cholecystokinin
CD	Circular dichroism
CHO	Chinese hamster ovary
Cha	Cyclohexylalanine
CREB	cAMP response element-binding protein
CRH	Corticotropin-releasing hormone
Cys(Bzl)	Boc-S-benzyl-l-cysteine
DIO	Diet-induced obese
DMN	Dorsomedial hypothalamic nucleus
ERK	Extracellular signal-regulated protein kinase
GFAP	Glial fibrillary acidic protein
GIR	Receptor induced by glucocorticoids
Glu(Obzl)	Boc-l-glutamic acid 1-benzyl ester
GLP-1	Glucagon-like peptide 1
GPCR	G-protein coupled receptor
GPR10	Receptor for prolactin-releasing peptide (known as hGR3 or rat ortholog UHR-1)
hArg	Homoarginine
HB	Hindbrain
HIPP	Hippocampus
His(Bzl)	Nα-Fmoc-N(im)-benzyl-l-histidine
HPA	Hypothalamic-pituitary-adrenal
HYP	Hypothalamus
Iba1	Ionized calcium-binding adapter molecule 1
ICV	Intracerebroventricular
JNK	C-Jun N-terminal protein kinase
KO	Knockout
LHA	Lateral hypothalamic area
MAPK	Mitogen-activated protein kinase
ME	Medulla oblongata
MEG-FA	Multiple ethylene glycol-fatty acid
MIB	Midbrain
MSG	Monosodium glutamate
myr	Myristic acid
NAc	Nucleus accumbens
1-Nal	1-napthylalanine
2-Nal	2-napthylalanine
Nle	Norleucine
Nme-Arg	N-methylarginine
NMR	Nuclear magnetic resonance
NPAF	Neuropeptide AF
NPFF	Neuropeptide FF
NPFF-R2	Receptor 2 for neuropeptide FF (known as GPR74 or HLWAR77)
NPY	Neuropeptide Y
NPY-1R	Neuropeptide Y receptor 1
NTS	Nucleus of the solitary tract
OF	Olfactory bulb
OLETF	Otsuka Long-Evans Tokushima Fatty
palm	Palmitic acid
P	Pituitary
PB	Parabrachial nucleus
PCR	Polymerase chain reaction
PEVN	Periventricular hypothalamic nucleus
PheCl_2_	(3,4-dichlor)phenylalanine
PheF_5_	pentafluoro-phenylalanine
PheNO_2_	(4-nitro)phenylalanine
Phg	Phenylglycine
PI3K	Phosphoinositide 3-kinase-protein kinase
PI3K-Akt-mTOR	Phosphoinositide 3-kinase-protein kinase B/Akt-mammalian target of rapamycin
PKA	Protein kinase A
PKC	Protein kinase C
PrRP	Prolactin-releasing peptide
PVN	Paraventricular hypothalamic nucleus
PS1	Presenilin 1
PTX	Pertussis toxin
RT	Reticular nucleus of the thalamus
SAR	Structure-activity relationship
SC	Subcutaneous
SHR	Spontaneously hypertensive rats
SHROB	Spontaneously hypertensive obese rats
SLM	Stratum lacunosum-moleculare
SON	Supraoptic nucleus
TH	Thalamus
TTDS	1,13-diamino-4,7,10-trioxadecan-succinamic acid
UCP-1	Uncoupling-protein 1
UHR-1	Rat ortholog of GPR10
VMN	Ventromedial hypothalamic nucleus
VRT	Ventrolateral reticular nucleus of the thalamus
WKY	Wistar Kyoto
WT	Wild-type
ZDF	Zucker diabetic fatty
7TM	Seven-transmembrane-domain receptor
